# Exploring human trainability: Design and rationale of Studies of Twin Responses to Understand Exercise as a Therapy (STRUETH) study

**DOI:** 10.1016/j.conctc.2020.100584

**Published:** 2020-06-09

**Authors:** Channa E. Marsh, Hannah J. Thomas, Louise H. Naylor, Katrina J. Scurrah, Daniel J. Green

**Affiliations:** a School of Human Sciences (Sport Science, Exercise and Health), The University of Western Australia, Perth, Western Australia, Australia; b Twins Research Australia, Centre for Epidemiology and Biostatistics, Melbourne School of Population and Global Health, The University of Melbourne, Victoria, Australia

**Keywords:** Twins, Exercise response, Exercise modality, Cardiovascular, Cerebrovascular

## Abstract

**Background:**

Exercise confers myriad health benefits and physical inactivity is a modifiable risk factor for many non-communicable chronic diseases. However, individual responsiveness to guideline-based exercise programs is idiosyncratic for health and fitness outcomes. It is not known whether the response of individuals to distinct exercise modalities tend to be concordant or whether there is a genetic contribution to variation in exercise responsiveness.

**Methods/design:**

Healthy, young adult (16–40yrs) monozygotic (MZ) and dizygotic (DZ) twin pairs were recruited and randomly assigned to 3 months of endurance or resistance exercise training. Twin pairs trained together. After 3 months of training in their randomly assigned mode, a washout period of 3 months was observed before twin pairs crossed over to complete 3 months of the alternate exercise intervention. Measures of cardiac morphology and function, cerebrovascular function, cognitive performance, peripheral artery function, biochemistry, blood pressure, body composition, skeletal muscle strength and cardiopulmonary fitness were collected before and after each exercise intervention (i.e. at weeks 0, 12, 24 and 36).

**Discussion:**

We adopted exercise modalities that produce distinct haemodynamic and physiological stimuli for physiological adaptation and recruited MZ and DZ twin pairs to address questions such as; *do individuals exhibit concordant responses to distinct exercise modalities?* and *what is the genetic contribution to adaptation resulting from distinct training modalities?* The results of this study will provide insight into the genetic and environmental contribution to exercise response to distinct modes of training, with implications for determining the optimal approaches to exercise prescription.

## Abbreviations

MZ: monozygotic
DZ: dizygotic
END: endurance training
RES: resistance training
UWA: University of Western Australia
MRI: magnetic resonance imaging
VO_2_: oxygen uptake
HR: heart rate
RPE: rating of perceived exertion
1RM: one repetition maximum
BP: blood pressure
TEE: total energy expenditure
AEE: active energy expenditure
SBP: systolic blood pressure
DBP: diastolic blood pressure
CCA: common carotid artery
FMD: flow-mediated dilation
GTN: glyceryl trinitrate
PWA: pulse wave analysis
PWV: pulse wave velocity
LV: left ventricle
RV: right ventricle
EDV: end-diastolic volume
ESV: end-systolic volume
LVID: left ventricular internal dimension
PLAX: parasternal long-axis
PSAX: parasternal short axis
ASE: American Society of Echocardiography
A4C: apical four-chamber
LA: left atria
A2C: apical two-chamber

## 1 Introduction

Physical inactivity is a crisis that is responsible for 6–10% (>5 million deaths) of annual global mortality from non-communicable diseases such as coronary heart disease and stroke. It caused 9% of premature mortality, or more than 5.3 million of 57 million deaths, worldwide in 2008 [1]. Unlike most commonly prescribed medications which target specific health outcomes, exercise can positively influence a wide range of important health determinants (weight control, cardiovascular health, cerebrovascular health, cognition) that contribute to preventing development of chronic diseases [2]. In spite of the well-established role of exercise and physical activity in prevention of chronic disease, recent studies suggest that up to 30% of individuals may fail to exhibit beneficial physiological responses to exercise interventions that emulate guideline recommendations for health [3]. These individuals can be considered “low-responders” for particular health parameters, in response to the type of exercise performed. This phenomenon was recently confirmed for the impacts of training on arterial health [4] and the idiosyncratic response of cardiorespiratory fitness (VO_2_) to endurance training has also been recently summarised [5].

In the context of responsiveness to exercise, a study from our laboratory suggested that health-related benefits may be modality specific. Spence et al. (2011) utilised two dichotomous exercise intervention modalities, endurance (END) and resistance (RES) training, which induce distinct haemodynamic and physiological stimuli for physiological adaptation. We found that cardiac adaptation differed between modalities [[6], [7], [8], [9]]. However, subjects in this study were randomised to parallel groups and completed only one exercise intervention/modality. It is therefore possible that the distinct responses to alternate exercise modalities that we observed might have been biased by the randomisation process. A within-subject cross-over design is a more appropriate approach to address the question of whether the response to distinct exercise modalities differs within individuals. Using this design, we can also determine whether individuals demonstrate concordant or discordant responses to distinct forms of training (i.e. whether low-response to one form of exercise training necessarily implies low-response to all modalities). This has implications for the optimisation of exercise prescription in humans, whereby switching to an alternate form of exercise could “rescue” subjects who otherwise may not have benefited.

In addition to the responder/non-responder phenomenon, and the question of whether low-response to one form of exercise training necessarily implies low-response to all modalities, a question relates to genetic predisposition to adaptation. Classical twin studies utilising monozygotic (MZ) and dizygotic (DZ) twin pairs have long been used to assess the contribution of unmeasured genetic and environmental effects on variation in measured traits [10,11]. Since MZ twins theoretically share 100%, and DZ twins ~50%, of their DNA [12] then a phenotype that is purely explained by genetics would hold a ratio of 2:1 consisting of correlations of 1 for MZ and 0.5 for DZ [13]. If the 2:1 ratio remains the same but the correlations are lower than these values, then both shared and unshared components may be influencing results [14,15]. In this way, classical twin studies allow researchers to stratify the total variance of a phenotype. Any excess correlation observed for MZ compared with DZ pairs is assumed to be due to the extra genetic material shared by MZ twins (under the equal environments assumption [16]), and the amount and proportion of variation in a measured trait due to additive genetic effects (A), shared environmental effects (C) and unshared effects (E) can be determined. The proportion of the total variation which is due to A is often referred to as the heritability [12,17]. Alternately, in cases where genetic and environmental effects cannot be separated, the use of a classical twin study design may make it possible to estimate the extent of familial aggregation [18].

The heritability of a phenotype has typically been assessed cross-sectionally, without any intervention [[19], [20], [21], [22], [23], [24], [25], [26], [27]]. The majority of studies purporting to assess the impact of heritability on the response to exercise have not, in fact, exercise trained individuals. Others have only utilised a single training modality, or focussed on discrete outcomes such as VO_2_ in twin or family studies [[28], [29], [30]]. Only a handful of exercise studies have assessed the heritability of additional outcome measures, including a study by our team that investigated peripheral vascular function and health in MZ and DZ twin pairs in response to an END training program [24,31]. Some additional outcomes in twin and family studies are examined in a systematic review and meta-analysis [32]. However, there are no studies, to our knowledge, that have utilised MZ and DZ twin pairs to assess the heritability of response to two distinct exercise modalities. The novel aspect of the present study design is that twin pairs trained together, using matched training intensity, so that each individual within a twin pair received an identical stimulus across the same time period. We also collected data on multiple health and fitness outcomes.

This paper describes the rationale and methodological aspects of a randomised controlled cross-over trial aimed at investigating the idiosyncratic nature and heritability of responses to distinct exercise training modalities (‘END’ and ‘RES’ exercise) in twin pairs. If exercise is considered medicine, then this study assesses the human response to different prescriptions, so that in the future, specific modes of exercise can be tailored on the basis of individualised health and/or fitness benefits.

## 2 Methods/design

### 2.1 Objectives

1. Primary objective: Assess the contribution of genetic and environmental effects on variation in cross-sectional data, and END and RES training responses, for each outcome measure
2. Secondary objectives:
  a. Assess whether individual response to training differs between modalities for each outcome measure
  b. Assess whether the concordance between response to END and RES modes of training will differ between individuals

### 2.2 Hypotheses

1. The heritability of response to training will differ according to whether cross-sectional or training data are assessed.
2. Response to exercise will be modality specific for each outcome measure.
3. Concordance between response to END and RES modes of training will differ between individuals.
4. Concordance between twins in a pair in terms of the response to END or RES modes of training will differ between MZ and DZ pairs.

### 2.3 Participant recruitment

**Fig. 1:**
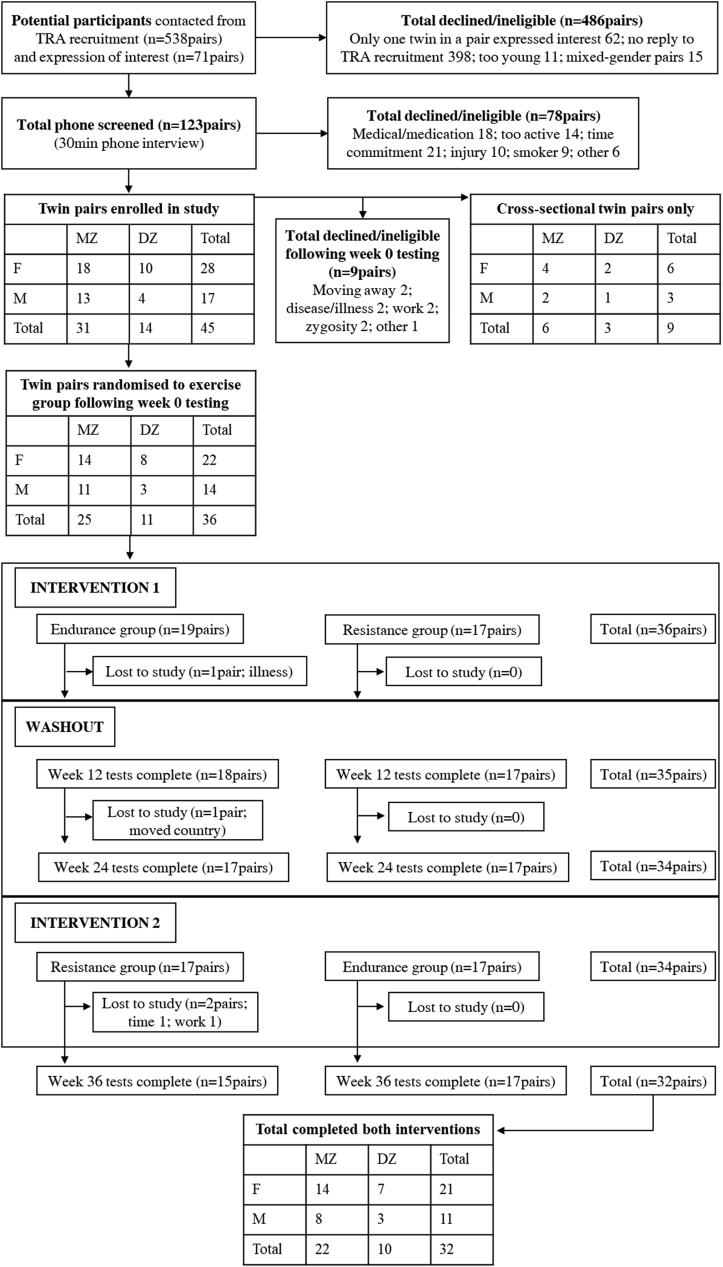
Participant recruitment, inclusion/exclusion, and intervention numbers. *TRA, Twins Research Australia; F, female; M, male; MZ, monozygotic; DZ, dizygotic.*

Healthy, same-sex DZ and MZ twin pairs, between the ages of 16–40yrs, were recruited to participate in the study. Recruitment started in September 2016 and was facilitated by Twins Research Australia (formerly Australian Twin Registry [33]). Twin pairs in the Metropolitan Perth-based region were sent emails, newsletters and conventional mail regarding participation in the study. Other recruitment methods included advertising in local and Perth-based newspapers, online and social media recruiting, The University of Western Australia (UWA) email lists, flyers around UWA and nearby establishments, and word of mouth. Baseline demographic data is displayed in Table 1, and a participant flow chart in Fig. 1.

**Table 1:** Baseline characteristics of participants enrolled in the study.

		Monozygotic (MZ)	Dizygotic (DZ)
		n = 62 (31 twin pairs, 18 F, 13 M)	n = 28 (14 twin pairs, 10 F, 4 M)
		Mean (SD)	Mean (SD)
Age (yrs)	F	24.2 (5.0)	23.8 (4.2)
	M	28.0 (5.9)	22.9 (5.3)
	Total	26.2 (6.1)	23.5 (4.5)
Height (cm)	F	170.2 (4.0)	168.6 (5.9)
	M	180.8 (4.5)	178.0 (6.6)
	Total	174.5 (6.5)	171.8 (7.5)
Weight (kg)	F	67.0 (19.6)	59.3 (9.4)
	M	81.0 (12.8)	69.1 (11.3)
	Total	74.9 (18.9)	62.6 (10.9)
BMI (kg/m^2^)	F	23.0 (6.1)	20.9 (2.8)
	M	24.7 (3.6)	21.7 (2.7)
	Total	24.4 (5.3)	21.1 (2.9)

### 2.4 Inclusions/exclusions, baseline testing and randomisation

**Fig. 2:**
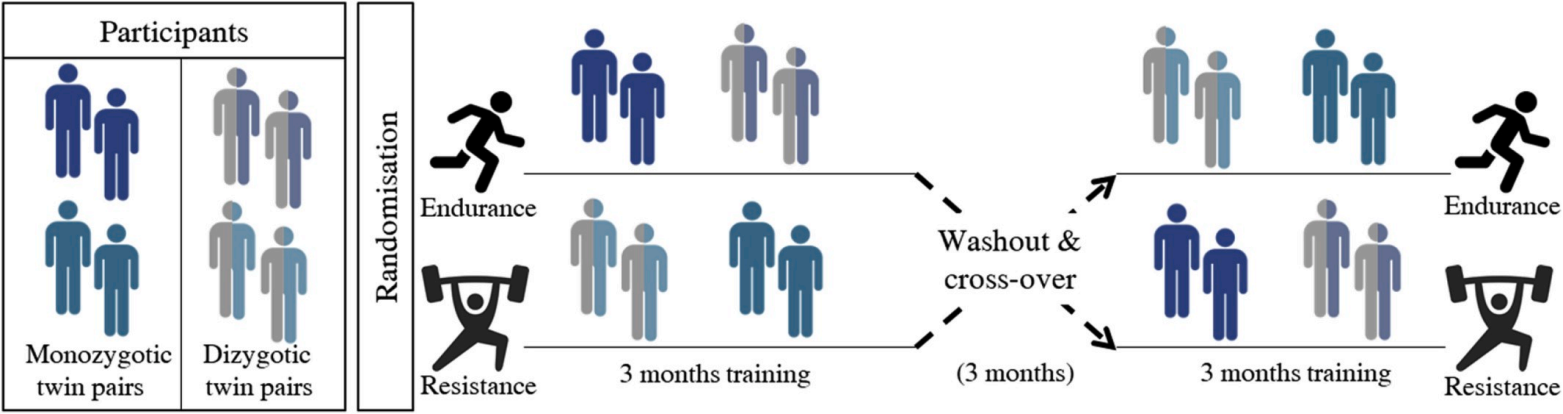
Study design schematic illustrating the longitudinal randomised cross-over exercise intervention. Monozygotic (single/solid colour) and dizygotic (multicolour) twin pairs are randomised to simultaneously participate in a 3 month supervised program, consisting of either resistance or endurance training. After a wash out period (3 months), they cross-over to complete 3 months of the alternate modality. (For interpretation of the references to colour in this figure legend, the reader is referred to the Web version of this article.)

**Fig. 3:**
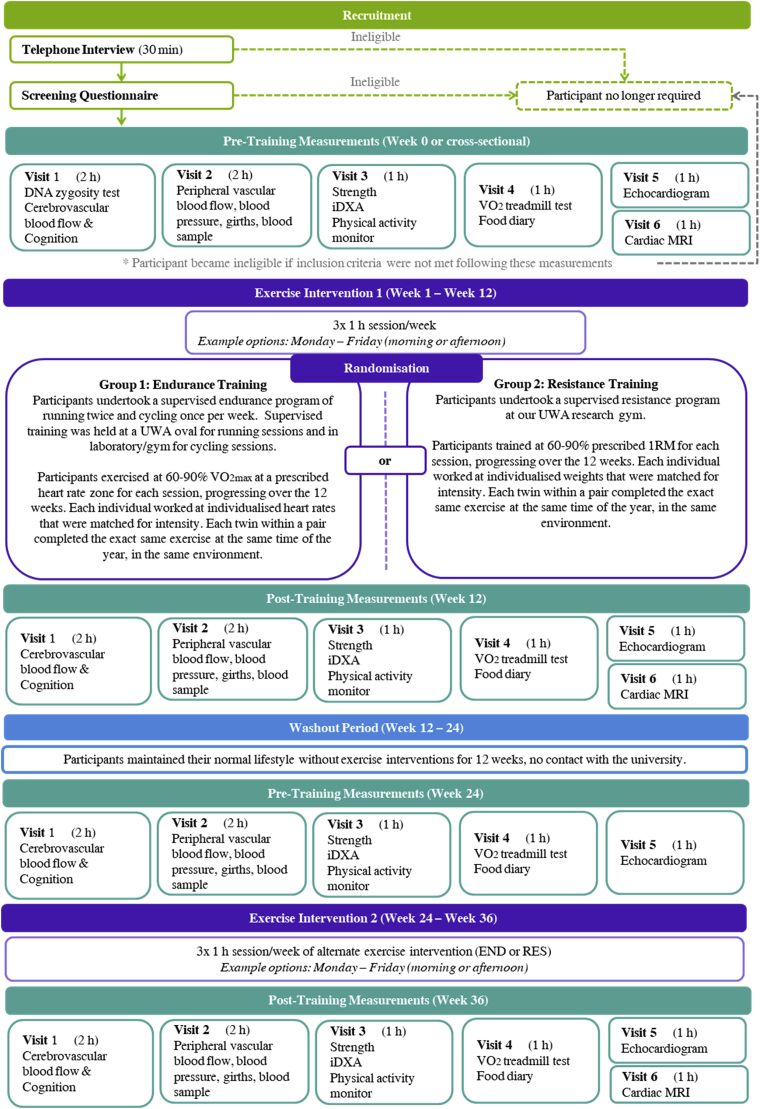
Twins and exercise study structure and timetable.

The study design is illustrated in Fig. 2. Participants were required to be apparently healthy, having no signs or symptoms of congenital heart disease, ischaemic heart disease, atrial fibrillation, stroke, kidney disease, liver disease, diabetes, epilepsy, respiratory disease, exercise-induced asthma. Resting systolic (SBP) and diastolic (DBP) blood pressure had to be < 160 and < 100 mmHg, respectively, with BMI ≤ 35. Subjects were excluded if they were smokers, or had been a smoker within the last 12 months, had severe mental illness or joint, muscular or spinal injuries/disease that would prohibit intense exercise. Any serious illness that would compromise survival (e.g. cancer) was an exclusion. Subjects were also required to be untrained, defined as doing less than the minimum Australian guidelines for physical activity recommendations (<150 min week of organised exercise). They were excluded if taking any prescribed medications for previously mentioned illness/disease that would affect outcome measures of the study. Subsequent to a successful phone interview and screening, participants attended six separate testing sessions (ranging from 45 min to 2 h duration; see Fig. 3), which made up the cross-sectional portion of the study and were used as additional screening for inclusion in the subsequent longitudinal training study. Using a website (www.randomization.com), twin pairs were randomised to either END or RES training to complete their allocated exercise together as a twin pair. That is, both twins in a pair completed the same exercise modality at the same time at matched relative exercise intensities. Completing the same exercise intervention together ensured that the stimulus of the exercise was identical and time- and environment-related factors that could impact physiological adaptation (i.e. time of the year, holidays, heat of the day) did not impact on the results within a pair. This is especially important in a twin study when trying to tease apart genetic, shared and individual environmental factors as the research environment created should be as concordant as possible within each twin pair.

### 2.5 Study design

This study was approved by the UWA Human Research Ethics Committee (reference number RA/4/7031) and was prospectively registered as a clinical trial (ACTRN12616001095459) in August 2016. Baseline testing commenced in November 2016, and the exercise intervention data collection was completed in September 2019. Oral and written consent were obtained from each participant prior to participation in the study. This study conformed to the standards set by the *Declaration of Helsinki*.

The study involved baseline screening and testing of each twin within a pair. Following this, twin pairs were randomised together to participate in either the 3 month END or RES training intervention. Both twins within a pair trained at matched relative intensities for each exercise modality. Participants were tested within 2 weeks of finishing the exercise intervention. Participants then entered into a 3 month washout period where they were instructed to return to their usual level of activities as well as maintain their usual diet. Following the washout period, participants returned for pre-testing prior to crossing over and completing 3 months of their second, alternate, exercise intervention (RES or END). Again, participants post-training measures were finished within 2 weeks of exercise intervention completion (Fig. 2). Participants within a pair completed all pre- and post-assessments within 1 week of each other and completed their exercise interventions in the same time period.

Experimental measures included body composition assessment, cardiorespiratory fitness, muscular strength, physical activity, food diaries and appetite-related questionnaires, biochemistry analysis, peripheral vascular assessments, pulse wave analysis and velocity assessments, cerebrovascular assessments, cognitive testing, sleep, anxiety and general health questionnaires, cardiac magnetic resonance imaging (MRI), echocardiograms, girth measurements and resting blood pressures (Fig. 3).

Throughout the duration of the study, participants were instructed to maintain their current lifestyle as much as possible, that is, to continue their current diet and exercise routine in addition to the three exercise sessions per week performed at UWA as part of the study. Compliance was assessed using activity monitors and food diaries. This maintenance was to ensure that changes throughout the study were due to the exercise intervention, rather than external influences.

### 2.6 Exercise training protocols

Exercise interventions were centre-based and supervised by a qualified sport scientist or an accredited exercise physiologist (AEP). Sessions were either in the morning or late afternoon/evening to avoid the heat of the day and to accommodate participant work schedules. Participants attended three exercise sessions a week at UWA. A standardised warm up and cool down were conducted in each session. If an injury prevented an individual from completing their prescribed exercise, then that individual and their twin were to complete a modified exercise instead. This ensured that twin pairs were receiving equivalent physiological stimulation throughout the study. Adherence to the training program was required to be 80% or higher across both interventions in order for the twin pair to be included in final analyses.

#### 2.6.1 Endurance (END) training

The two exercise modalities (END and RES training) were chosen as they provide distinct haemodynamic and physiological stimuli for adaptation. The exercise interventions were intensity matched and progressively overloaded throughout the 12 week programs.

Training intensity was personalised and matched within the twin pair for each session and monitored via continuous heart rate (HR) assessment. Target HRs were calculated from HR at a percentage of the initial graded exercise VO_2_max test, so that they were individualised, but matched, for intensity between individuals and within-twin pairs. During each exercise session, intensity was monitored continuously using a HR monitor (Polar RS300X HR monitor, Polar Electro Oy, Finland) and recorded at regular intervals. This allowed the trainer to ensure participants were working at their specified HRs for each session. Participants completed two running sessions and one cycling session per week. Running sessions took place on the UWA 200 m grass track, and cycling sessions took place in the UWA laboratory/gym on cycle ergometers (Ergomedic 828 E, MONARK, Vansbro, Sweden). Duration and intensity progressively increased over the course of the 3 month exercise intervention from 15 min, to 60 min of exercise.

The 12 week END program followed a periodised progressive macrocycle plan consisting of three mesocycles of 4 weeks each. The first mesocycle, the ‘general preparatory’ phase (weeks 1–4), consisted of low training intensity/volume (lots of walking/jogging at 60% HR, 1–2.5 km running and/or 15–25 min cycling/running) including a long warm up focusing on running drills, technique and dynamic stretching. The second mesocycle, the ‘high intensity’ phase (weeks 5–8), consisted of higher intensity work with higher HRs (up to 90%), and distance/duration (2.5–5 km running and/or 25–40 min cycling/running). The final mesocycle, the ‘maintenance and distance’ phase (weeks 9–12), consisted mostly of maintaining sub-threshold HRs (70–85%) for longer distance/duration (5–7 km running and/or 40 min in week 9–60 min by week 12 cycling/running). In the mesocycles, a 1:1 structure loading existed with a ‘hard’ loading week followed by an ‘easier’ or ‘maintenance’ week. Within each week there existed a structured load with a ‘harder’ run session, an ‘easier’ run session and the cycling session was always the longest duration session in each week, adapting many principles from previously published work [8,34].

#### 2.6.2 Resistance (RES) training

Number of repetitions, number of sets, weight and rate of perceived exertion (RPE) were recorded for every session to allow the trainers to monitor the work load undertaken by the participants. The main purpose of this monitoring was to ensure individual workload was equal within twin pairs. Specific exercises, number of sets and number of repetitions was standardised across all participants. However, individual weights were prescribed from each participants pre-training one repetition maximum (1RM). The 12 week RES training program also followed a periodised plan consisting of four mesocycles of 3 weeks each, as shown in Table 2. The first mesocycle focused on muscular endurance (weeks 1–3) where intensity was low (60–70% 1RM), reps were high (12–15 reps) and rest periods between sets was short (30–60s). This first few weeks allowed participants to focus on forming good technique habits, condition muscles and assist recovery between sessions. The second mesocycle was muscular hypertrophy (weeks 4–6) where intensity increased (70–75%), reps decreased (10–12 reps), but rest periods remained short between sets (30–90s). Weeks 7–9 was a progressive step between muscular hypertrophy and strength where intensity increased (75–80%), reps decreased (8–10 reps) and rest periods between sets increased (1–2 min). The last mesocycle focused on improving muscular strength where intensity increased (80–90%), reps decreased (5 reps) and rest periods between sets increased (3–5 min).

**Table 2:** Resistance training breakdown of mesocycles.

Weeks	Training Type	Intensity (%1RM)	Repetitions	Rest
1–3	Muscular Endurance	60–70%	12–15	30–60s
4–6	Hypertrophy	70–75%	10–12	30–90s
7–9	Hypertrophy/Strength	75–80%	8–10	1–2 min
10–12	Strength	80–90%	5	3–5 min

Each session focussed on one of the five main exercises (two upper body – bench press and standing military press; three lower body – squats, deadlift and leg press) that were rotated alternating upper and lower body on separate days. There were secondary exercises performed during each session that used muscle groups that were similar to, or would assist in performing, the main exercise of the session (i.e. staggered feet leg press, seated row, lat pulldown). Participants performed a standardised warm up before completing their session and a standardised 5 min core exercises and cool down at the end of the session. To guide participants progressions, 1RM assessments were repeated half way through their 12 week program.

### 2.7 Outcome measures

#### 2.7.1 Zygosity DNA testing

A DNA cheek swab was obtained following written consent during the participants’ first testing session to determine zygosity of twin pairs for inclusion in the study (EasyDNA AU, Springwood, QLD). Four swabs were taken in total, two swabs on each side of the participants mouth, rubbed ten times against the inside of the participants cheek. Participants collected, packaged and sealed their own samples to eliminate the risk of cross-contamination. Samples were sent to an independent genetics laboratory for testing of zygosity as the probability based on genetic markers of twin pairs being either MZ or DZ. Participants elected whether they would receive the results of the DNA test. When initially recruiting for the study twin pairs self-reported their zygosity, however when they were DNA zygosity tested 10 self-reported DZ twin pairs were actually found to be MZ. Therefore, DNA zygosity testing was a major strength within the current study as heritability is determined based on zygosity. Nearly 25% of twin pairs self-reported zygosity was incorrect in our study and our results could have been very different if DNA samples had not been acquired. A systematic review and meta-regression has also concluded that self-reported rather than DNA tested zygosity has a significant effect on heritability results [35].

#### 2.7.2 Cardiorespiratory fitness

Cardiorespiratory fitness was assessed via a graded exercise treadmill test repeated at the same time of day for each repeat visit for a given individual, and was conducted by the same researcher. At baseline, this exercise test served the dual purpose of being a fitness test and a screening tool for inclusion in the training study, along with providing prescriptive information for the END training intervention. For the 2 h prior to the exercise test, participants were instructed to abstain from food and drinks other than water. Participant height (cm) and weight (kg) were recorded and they were then asked to stand on the treadmill. The graded exercise test protocol and Borg RPE scale [36] was explained to the participant. An automated blood pressure (BP) cuff (Dinamap V100, GE, Healthcare, USA) was fitted to the left arm and a HR monitor (Polar RS300X HR monitor, Polar Electro Oy, Finland) was fitted for recording of resting (upright) HR. Resting blood lactate was then extracted from the earlobe of the participant using a lancet, collecting the second droplet of blood in a lactate pro 2 (Lactate Pro2 LT-1730, Akryay USA). The participant was then fitted with a headframe, Hans Rudolph mouthpiece and nose clip. The two-way mouthpiece permitted subsequent analysis of continuous expired air using a metabolic cart (Parvomedics TrueOne 2400, Salt Lake City, UT) which was calibrated according to manufacturer specifications, using gases of known concentrations with a 3.0 L syringe prior to each test. The reliability for this cart (tested ~7 days apart) has been tested to produce a VO_2_max technical error of 0.15 L min, or 2.4%. Baseline resting steady state respiratory data (2 min) was collected with the participant standing stationary on the treadmill prior to commencing the graded exercise test. The treadmill starting speed was set at 8 kph and increased by 1 km/h each stage, while the gradient stayed at 1% throughout the test. Each stage was 3 min long, interspersed with a 1 min rest period. In the last 10s of each stage, HR was recorded and at the end of each stage, participants were instructed to brace their hands on the side railings of the treadmill and place feet on the stationary sides. This started the 1 min rest period between each stage where speed was immediately decreased to 2 km/h. A blood lactate and RPE for that stage were recorded before participants were instructed to step back onto the treadmill to walk at 2 kph until the 1 min rest period was complete whereupon the treadmill speed was then increased to the speed of the next stage. Standardised verbal encouragement was provided throughout the test to elicit maximal effort. The test was completed once the participant reached volitional exhaustion. Participants were able to terminate the test at any time by bracing and jumping off the treadmill or signalling to the researcher they wanted to stop.

#### 2.7.3 Muscular strength

Maximal muscular strength was assessed according to a 1RM protocol, before and after each exercise intervention, as well as half way through the RES intervention so that exercise intervention weights could be appropriately progressed (for each individual within a twin pair). The reliability for upper and lower body 1RM performed ~7 days apart in healthy subjects has provided a high ICC (ICC > 0.99) and high correlation (r > 0.9) [37]. Participants arrived at the gym at the same time of day for each repeated testing session. Participants performed 1RM tests of bench press and leg press, conducted by the same researcher at each visit. These tests were chosen as they are considered relatively safe when performed with assistance of a qualified sport scientist for untrained individuals and incorporate the major muscle groups for both upper and lower body strength. Prior to participants starting their tests, the researcher demonstrated and explained correct lifting technique. The 1RM protocol included a warm up (five reps of 20 kg for bench press and ten reps of 60 kg for leg press), followed by five attempts to reach maximal muscular contraction in one repetition. There were 3 min breaks between efforts and weight was added depending on the participants reported RPE after each attempt. This process was repeated until the participant reported an RPE of 20, they failed to complete their repetition with correct technique, or the researcher deemed it necessary. Standardised encouragement was performed by the researcher to all participants during the test.

#### 2.7.4 Physical activity

Total (TEE) and active (AEE) energy expenditure were measured throughout the study, pre-post each exercise intervention (weeks 0, 12, 24 and 36). The primary purpose of this was to examine whether the results obtained from the study were due to the exercise interventions that were conducted throughout the study, or external and incidental changes in TEE and AEE that occurred outside of the planned training sessions. Actiheart monitors (Actiheart 4, CamNtech Ltd, Cambridge, UK) were used to assess TEE and AEE as they include a combined HR and accelerometer, which have been shown to be valid in adolescents [38]. Participants were familiarised with the Actiheart monitors for use, care, skin preparation and instructed to wear the Actiheart for a period of 5 days, and not to wear whilst showering. They were also instructed to take the Actiheart off for sleep (“put Actiheart on first thing when they get up and take off when they get in to bed”) as we were interested in estimating TEE and AEE of participants normal daily activities. Actihearts were first placed on participants with two ECG electrodes (40 mm × 34 mm; AgCl, Red Dot 2560, 3 M) either above or below the nipple line with the medial electrode placed in the centre of the chest (usually on the sternum) and the lateral electrode placed horizontally to the left side with the wire straight but not taut. A signal test was first performed to determine the best positioning of the electrodes for each participant where a HR trace is best conducted. Electrode positioning was noted and repeated at subsequent timepoints. Following determination of the best site for each individual to wear their Actiheart for the desired days, a long-term test was set up which activated continuous recording over 1 min epochs. The Actiheart data was downloaded after each trial using a reader interface unit and analysed using Actiheart software (Actiheart software 4.0.65, CamNtech Ltd, Cambridge, UK). The main outcome measures collected were TEE, AEE and quantification of time spent in sedentary, light, moderate or vigorous physical activity according to METS [39,40].

#### 2.7.5 Food diary and appetite

A food diary was administered to each participant along with some general trait appetite-related questionnaires (weeks 0, 12, 24 and 36) to complete the week prior to or immediately following each exercise intervention. Food diaries covered four consecutive days, with one having to be a weekend day, on participants ‘normal’ days of eating. Food diaries required participants to record the type, brand, cooking method, portion size and timing of ingested food and beverage in detail, for the purpose of assessing whether eating habits had remained constant throughout the study. Mean total daily energy intake, together with the quantity of carbohydrate, fat, and protein consumed were determined from these records using a commercially available software program (Foodworks 9; Xyris Software, Queensland, Australia). General appetite-related questionnaires included the food craving inventory – UK version (FCI-UK [41]), the compensatory eating motives questionnaire (CEMQ [42]), three factor eating behaviour questionnaire (3FEBQ [43]) and the trait general food cravings questionnaire (G-FCQ-TRAIT [44]). Participants were also administered two acute appetite questionnaires, the visual analogue scale (VAS) [45] and general food cravings questionnaire – state (G-FCQ-STATE) [44], at weeks 0, 12, 24 and 36 in a fasted state at the same time of the morning at each time point, and immediately prior to and after their graded exercise test. The VAS was also administered with the food diary and participants were required to complete a VAS for perceived appetite upon waking up in a fasted state, immediately prior to consuming lunch, dinner and going to bed.

#### 2.7.6 Body composition

Body composition was measured using dual energy X-ray absorptiometry (Lunar iDXA, GE, healthcare, USA). Standard calibration and quality assurance procedures were used, in line with the equipment documentation (www3.gehealthcare.com). Outcome measures included regional and whole-body percent fat; grams of lean tissue, fat tissue, bone mineral content, visceral adipose tissue; gynoid and android fat percentages and ratios and bone mineral density. Participants arrived at the laboratory at the same time for each of the four measurements (pre- and post-each exercise intervention), usually in the afternoon/evening. Participants were instructed to fast for 3 h, be normally hydrated (i.e. not hyper/hypo-hydrated, or post exercise) and eliminated prior to the test. The inter- and intratester reliability results of our machine are very highly repeatable (ICC >0.99) in 52 tested participants [46].

#### 2.7.7 Biochemistry

Participants arrived at the laboratory at the same time each morning for repeated measures following an overnight fast. Participants had refrained from any moderate/vigorous physical activity, alcohol and medication the 24 h prior to the testing session. This was confirmed at the start of each session verbally and recorded on the testing sheet. Assessments were conducted in a temperature, light and noise standardised room. These conditions were also adopted for the cerebrovascular blood flow and cognition, peripheral vascular blood flow, blood pressure and girths assessments. Blood was drawn from the antecubital fossa using a 21G needle by a trained phlebotomist in the UWA laboratory into five collection tubes (2× Vacuette by Greiner bio-one, Kremsmünster, AT; 3× Vacutainer, DB). Sterile techniques were applied, identical to those used in routine clinical services, and a maximum of 20 mL of blood was drawn per visit. Three blood tubes (2× lithium heparin, 1× fluoride oxalate) were analysed by a commercial pathology laboratory, for lipids (total cholesterol, triglycerides, low-and high-density lipoprotein and the ratio of total cholesterol: high-density lipoprotein), fasted blood glucose, insulin and a cardiovascular-related inflammatory marker (high-sensitivity C-reactive protein). Two tubes (serum – serum separator tube; plasma – EDTA) were collected and refrigerated for 20 min (so that the serum tube could clot) before being centrifuged together for 10 min at 2000 RCF at 4 °C. Resulting plasma and serum were pipetted and stored at −80 °C in 1 mL aliquots.

#### 2.7.8 Resting blood pressure

Supine resting blood pressures on the right arm were taken every 3 min over a total of 15 min using an automated sphygmomanometer (Dinamap V100, GE, Healthcare, USA). This was done in a quiet dark room with the subject alone, undisturbed and lying supine. Mean arterial BP, SBP, DBP and HR of the last two assessments were recorded.

#### 2.7.9 Cerebrovascular function

Cerebrovascular function was assessed by i). combining bilateral measures of cerebral artery blood flow velocity obtained using non-invasive, transcranial Doppler ultrasound techniques; and ii). cerebral artery diameter and blood flow velocity of the internal carotid and vertebral arteries using duplex ultrasound according to standardised approaches [47]. The reproducibility coefficient of variation of the resting assessment of middle cerebral artery velocity using transcranial Doppler was 4.5% from day-to-day within 71 subjects [48] and 1.5% for internal carotid artery diameter using duplex ultrasound for within-day variability [49]. In addition to resting baseline intra-cranial velocity and extra-cranial velocity and diameter recordings, four test conditions were implemented to challenge the cerebrovascular system and provide information relating to functional responsiveness to distinct types of stimulation. These four tests reflected cognition, dynamic cerebral autoregulation, cerebrovascular CO_2_ reactivity and exercise (described in more detail below; Supplementary Fig. 1). Intra-cranial velocity and extra-cranial velocity and diameter, as indices of cerebral blood flow, were assessed.

At the start of the session, the participant was fitted with a head frame (Marc 600, Spencer Technologies, Seattle WA, USA), with a 2-MHz ultrasound probe mounted on each temple, to facilitate imaging of the middle cerebral artery (right) and the posterior cerebral artery (left). A Finometer (Finometer Pro, Finapres Medicak Systems, Amsterdam ZO, The Netherlands) allowed continuous monitoring of beat-to-beat blood pressure via photoplethysmography. A three lead ECG was attached to monitor HR (BIO Amp CF, ADInstruments, New South Wales, Australia). Following instrumentation, the participant was seated on a semi-supine bench (incline of bench remained consistent to allow optimal reproducibility of blood vessel images) where the procedure was explained to them in detail. Once the participant was in position, a mouthpiece connected to a spirometer (Spirometer, ADInstruments, New South Wales, Australia) was placed in the participants mouth, with a nose clip, in order to measure expired gases (Gas Analyzer, ADInstruments, New South Wales, Australia) during baseline measures and all four testing conditions. A 5 min resting baseline was recorded, followed by 1 min of resting vertebral and internal carotid artery baseline (LabChart 7 software, AD Instruments, Sydney, Australia). The following tests were then conducted, always in the same order and always by the same sonographer in a given twin pair.

##### 2.7.9.1 Cogstate

A battery of reliable and well-validated cognitive tests was administered to encompass a range of cognitive domains [50]. These tests were conducted by a trained researcher and test results were analysed by the same individual, blinded to testing order. The following tests were conducted:

• Detection test- A 3 min test which provides a measure of psychomotor function reaction time and is assessed by speed of performance.
• Identification test- A 3 min test which measures attention and choice reaction time via speed of performance.
• Maze learning test- A 7 min test that measures executive function via total number of errors made during the task.
• International shopping list test- A 5 min test that measures verbal learning and episodic memory. This is done by measuring the total number of correct answers in remembering the list on three consecutive trials, at a single session.
• Two back test- A 4 min test which measures working memory via on-line monitoring, updating and manipulation of remembered information. This test is scored using accuracy of performance.
• Set-shifting test- This test measures executive function or the ability to display flexibility using total number of errors in 7 min.
• Modified chase task- This 4 min test measures executive function or response inhibition using speed of performance.
• SF-12 health survey- This 12-item questionnaire takes approximately 2 min to complete and assesses health and well-being. Specific outcome measures include physical and social functioning, role limitations due to emotional problems, mental health, energy/vitality, pain, and general health perception.
• PSQI- This 5 min survey provides a measure of the participants current sleep behaviour.
• DASS21- This 3 min survey provides information on measures of depression, anxiety and stress.

##### 2.7.9.2 Dynamic cerebral autoregulation

For this test, cerebral blood flow was assessed in response to different ‘doses’ of BP manipulation. Oscillations in BP were stimulated by a squat-stand stimulus of two different frequencies. First, participants standing manual BP was taken, followed by a 1 min standing baseline, prior to commencing 3 min of squat stands at a frequency of 0.33Hz. After the commencement of this test the participant was seated, allowing 5 min recovery, before they commenced a replica procedure at 0.66Hz. This methodological approach is in accordance with published guidelines on this technique [51]. Impairment in dynamic cerebral autoregulation is strongly linked to adverse clinical outcomes [52,53].

##### 2.7.9.3 Cerebrovascular CO_2_ reactivity

This test measures a dose-response effect to different ‘doses’ of blood CO_2_, achieved by switching from room air to gas mixtures that contain differing concentrations of CO_2_. Impairment in cerebrovascular CO_2_ reactivity is an independent predictor of stroke risk and cognitive impairment [54,55]. The participant initially breathed room air for a 1 min baseline period before being connected to a Douglas bag containing a mixture of 3% CO_2_, 21% O_2_ and balanced N_2_. The participant continued to breath normally for 3 min, before the Douglas bag was disconnected, and the participant breathed normally for 2 min of recovery. The mouth piece was then removed, and the participant was given a 5 min break to allow full recovery before the next test. This process was repeated with a bag mixture of 6% CO_2_, 21% O_2_ and balanced N_2_.

##### 2.7.9.4 Exercise

This test is used to assess cerebral blood flow to differing intensities of exercise, achieved by cycling for 2 min at three different Wattage. The participant was positioned semi-supine on a bench with their feet on a Monark biycle (Ergomedic 828 E, MONARK, Vansbro, Sweden), with the mouthpiece in. A 1 min baseline was recorded before the participant commenced their first 2 min stage at 60 W, followed immediately by 2 min at 90 W and again by 2 min at 120 W. The participant maintained their RPM between 50 and 60RPM throughout the entire 6 min test.

#### 2.7.10 Conduit artery structure and function

A 10-MHz multifrequency linear array probe attached to a high-resolution ultrasound machine (T3300, Terason, Burlington, Massachusetts) was used to simultaneously assess brachial and femoral artery structure and function in accordance with the methods described by Thijssen in 2011 [56]. Common carotid artery (CCA) wall thickness and lumen diameter was also assessed.

##### 2.7.10.1 Carotid artery wall thickness

Following an initial 15–20 min rest period, resting wall thickness and diameter of the left and right CCA was obtained using the high-resolution ultrasound machine (Supplementary Fig. 2). 10s longitudinal B-mode recordings of the middle, anterior and posterior (in that order) views of the CCA were obtained approximately 2 cm proximal to the carotid bifurcation. Settings were adjusted to focus on the superior and inferior walls of the CCA arterial lumen interface and the intima media adventitia [57].

##### 2.7.10.2 Endothelium-dependent vasodilation: flow-mediated dilation

To assess endothelium-dependent vasodilation of the brachial and femoral arteries, the flow-mediated dilation (FMD) technique was used (Supplementary Fig. 3), described in more detail elsewhere [56]. The reliability of FMD tested ~7 days apart in healthy subjects has a high correlation (r = 0.82) [58]. In summary, a rapid inflation-deflation pneumatic cuff (D.E. Hokanson Inc., Bellevue, WA, USA) was wrapped around the left forearm, 2 cm distal to the olecranon process. A second larger cuff was positioned on the upper left thigh ~15 cm distal of the inguinal ligament, or ~5 cm proximal to the patella and patella tendon. The arterial-lumen interface was optimised for longitudinal B-mode images of the brachial and femoral arteries. Continuous Doppler pulse wave velocity of each artery was simultaneously obtained (by two expert vascular sonographers) using the lowest possible isonation angle (60°). Dynamic range and Doppler gain settings were consistent between machines, and specifications were replicated for each individual on repeat visits. The left arm was placed at the height of the heart in approximately 80⸰ abduction from the body. The arm was supported underneath the muscles and the hand slightly pronated in a relaxed position for the participant. The brachial artery was scanned in the middle or upper third of the arm, depending on positioning of the brachial artery bifurcation. The brachial artery was also scanned either through the biceps brachii or between the biceps and triceps brachii muscles, depending on which gave a cleaner image for analysis for that particular participant. The femoral artery was assessed in the proximal third of the thigh, at least 5 cm distal from the bifurcation, with the left leg in slight (<5°) lateral rotation. These anatomical specifications were noted at the initial testing session and replicated at the repeated visits.

A baseline scan was recorded for a period of 1 min to assess resting blood vessel diameter, shear rate and blood flow velocity. Both blood flow occluding cuffs were rapidly inflated to a suprasystolic pressure of 220 mmHg for 5 min. Recording resumed 30s prior to cuff deflation so that it wasn't missed and continued for 3 min post-deflation [59]. Peak artery FMD (absolute and %), shear stress, time to peak and artery diameter pre-post cuff were analysed as previously described [56,58,59].

##### 2.7.10.3 Endothelium-independent vasodilation: glyceryl trinitrate

Utilising the same machine settings, optimisation and placement of the ultrasound probes on the brachial and femoral arteries as those described above for the FMD assessment, endothelium-independent vasodilation was assessed following sublingual glyceryl trinitrate (GTN) administration. The reliability of GTN also has a high correlation (r = 0.84) [58]. Baseline recording of resting artery diameter, shear stress and blood flow velocity were conducted for 1 min. One sublingual dose (400 μg) of GTN was administered. Participants were also instructed to not swallow or talk for 1 min post-administration so that the medicine was absorbed into the blood stream across the mucosa, rather than being swallowed. Ultrasound measurements of artery diameter and blood flow velocity were recorded from 3 to 8 min post-administration. Analysis followed similar guidelines to that of FMD analysis.

##### 2.7.10.4 Localised ischaemic handgrip exercise-induced vasodilation

A custom-built hand grip device was moved into position with the participants arm supported in a comfortable position. Ischaemic handgrip exercise provides an index of maximal diameter and blood flow in the brachial artery [60]. This followed the same protocol of the FMD procedure in the arm. In addition, participants were instructed to handgrip to a metronome for the middle 3 min of cuff inflation (25 isotonic contractions per minute with the weight of the handgrip machine set at 1.25 kg). Peak artery FMD (absolute and %), blood flow, shear stress, time to peak and artery diameter, pre-post cuff deflation were analysed.

##### 2.7.10.5 Peripheral arterial stiffness and velocity

Peripheral artery stiffness and pulse wave velocity were assessed via SphygmoCor (SphygmoCor, AtCor Medical, Sydney, Australia. Model Xcel. Software version 7.01 S). The validity and reliability of the SphygmoCor Xcel has previously been investigated and described [61]. Pulse wave analysis (PWA) is assessed with participants instructed to lay flat with their eyes closed. A brachial cuff is placed on the left arm and three automated cuff inflations are acquired at 60s intervals over 5 min with an additional inflation at the end of the 5 min to capture 10s PWA of the brachial artery (AIx@75). In this study PWA was used to assess central blood pressure (pressure at the level of the aorta) and the stiffness of the brachial artery. Blood pressure was also derived at each of the three measurements and the average DBP and SBP was used for pulse wave velocity (PWV; Supplementary Fig. 4) assessment.

Carotid-femoral PWV indicates arterial stiffness, calculated by measuring the speed of the pulse travelling down the arteries. The brachial cuff was removed, and a femoral cuff was placed around the upper right thigh, as close to the groin as possible, with the lead on the middle lateral side of the leg pointing towards the head. While the right thigh was flexed, participants located their femoral crease with their index finger and the distance between the anterior femoral crease and proximal superior part of the femoral cuff was noted in the system. The right carotid pulse was located and marked at its most prominent point along with marking the sternal notch. The distance between the carotid pulse-sternal notch and the sternal notch-proximal superior femoral cuff was also recorded and noted. These indicate the PWV distance between the carotid and femoral pulses. The system was set to a capture time of 10 s, subtraction method was used, and cuff/tonometer sync was turned off. The femoral cuff was inflated around the right thigh to a pressure of 180 mmHg to capture the femoral pulse. Simultaneously, a tonometer was held on the carotid pulse marked to capture the carotid pulse. The PWV was automatically captured when ~10 corresponding carotid and femoral pulses were higher than the quality threshold. A second measure was repeated to ensure consistent results and if the difference between the first and second reading was >0.5 m/s then a third reading was performed.

#### 2.7.11 Cardiac measures

##### 2.7.11.1 Cardiac MRI

Measures of left (LV) and right (RV) ventricular morphology were assess using a 1.5T MRI machine (Magnetom Espree, Siemens, Erlangen, Germany) in a 30 min scan. The reliability of CMR preformed 7 days apart has reported high correlations for LV mass (r = 0.99), EDV (r = 0.99), ESV (r = 0.93) and EF (r = 0.94) with non-significant differences between timepoints (*P* > 0.05) [62]. Participants were scanned in the supine position with a posterior phased array spine coil and anterior flexible phased array body surface coil. Multi-plane breath-hold using steady-state free procession (TrueFISP) localisers were acquired to obtain standard cardiac imaging planes. For all sequences, the breath-hold times varied between 5 and 10s, depending on the participants HR. To evaluate functional parameters, breath-hold TrueFISP cine images were acquired using a retrograde ECG trigger covering the whole R-R interval. Images of the LV and RV were acquired in short axis plane, perpendicular to the ventricular septum. Between 10 and 12 slices were acquired (6 mm slices/4 mm gap, FoV 320–350 mm, TR = 37.68, TE = 1.29 flip angle 70–80 deg, resolution 256 × 166, BW 930). Cine images of the four-chamber and LV outflow tract were also acquired (6 mm slices, FoV 300–330 mm, TR = 38.28, TE = 1.32, flip angle 70–80 deg, resolution 224 × 224, BW930). All cardiac MRI analysis was performed using specialized software (ARGUS, Siemens). Analyses were independently repeated and confirmed by an experienced cardiologist, who was blinded to twin zygosity and pairing. To assess LV morphology, systemic and pulmonary outflow tracts were auto-segmented in individual slices on cine images and then manually adjusted where necessary. Short axis cine loops were inspected to define LV end-diastolic volume (EDV) and end-systolic volume (ESV) frames. The mitral valve markers were automatically displayed on two- and four-chamber cine views but could be manually manipulated if inconsistent tracking occurred throughout the slices. However manual placement was required for markers of the tricuspid valve in RV analysis. The basal LV and RV slices were taken as the first slice below the level of the mitral valve and tricuspid valve, respectively. Therefore volumes above the aortic valve and those surrounding the thin myocardial wall in the mitral valve and tricuspid valve plane were excluded from analysis. Endocardial and epicardial LV borders were automatically contoured (but could be manually manipulated if required), including the septum but excluding the papillary muscles which were added to LV EDV and LV ESV in accordance with the methods described previously [8,63]. LV mass was calculated by summing the LV EDV within the epi- and endo-cardial borders of the short-axis slices, multiplying the myocardial tissue volume by its specific density (1.05 g cm−3). The LV EDV and LV ESV were utilised to ascertain stroke volume, ejection fraction and cardiac output. The RV endocardium was manually traced on each short axis frame. Data was validated by checking that systemic flow, pulmonary flow, LV and RV stroke volume were all within 5% of each other. Any deviations resulted in re-analysis and, if required, review by a cardiologist. To facilitate valid comparison to previously obtained echocardiographic data, a representative measure of LV internal cavity dimension during diastole (LVIDd) and systole (LVIDs), as well as interventricular septum and posterior wall thickness were determined from the short-axis view between the LV outflow tract and papillary muscle slice. Any error >2% resulted in both measurements being re-measured at a later date by a blinded reviewer. For assessment of RV morphology, instructions for tracing the endo- and epicardial borders were taken from the methodology described previously [6]. To overcome difficulties in determining the RV in the basal two slices, short axis cine loops were inspected for end-systole, which was defined as the frame with the smallest ventricular cavity. In the most basal end-diastolic slice, a RV contour was drawn only if it was visible for a minimum of three-phases in diastole, whereas the visible RV contour was always traced in the most basal end-systolic slice. RV mass was determined by subtracting the volumes between the epi- and endocardial borders, multiplying by slice thickness and myocardial specific density and summing the slices of this area during end-diastole and end-systole. As the epicardial border overlapped both the septal part of the endocardial border and at the valve planes, RV mass was calculated from the RV lateral wall only. RV EDV and ESV, as well as derived stroke volume, ejection fraction and cardiac output were calculated.

##### 2.7.11.2 Echocardiography

All echocardiographic images were acquired on a commercially available ultrasound system (EPIQ-7, Koninklijke Philips N.V., Philips Electronics Australia Limited, New South Wales, Australia) using a 1–5 MHz phased array transducer. A highly experienced qualified sonographer performed all assessments and was blinded to exercise group allocation. Subjects lay in the left lateral decubitus position and image acquisition followed standard adult transthoracic echocardiographic protocol [64]. Two-dimensional images of the parasternal long-axis (PLAX) and the parasternal short axis (PSAX) views were obtained according to the American Society of Echocardiography recommendations (ASE [[64], [65], [66]]). Images of the ventricles and atria were obtained from an apical four-chamber (A4C) view, with the walls and septa of each chamber achieved for assessment of size and function [64]. LVIDd, LVIDs, septal and posterior wall thicknesses were determined from the PLAX view, while left atrial (LA) and LV chamber volumes were determined from the A4C and apical two-chamber (A2C) views. The function of the mitral, tricuspid and aortic valves were also obtained in the apical view using colour and spectral Doppler. Diastolic function of mitral inflow, and annular tissue Doppler of medial and lateral mitral annulus for diastolic function, was assessed using pulsed Doppler and tissue Doppler in the A4C view [66]. With the participant laying supine with knees bent, the subcostal view was obtained by pressing the transducer into the area just inferior to the sternal xiphoid process. This view was used to investigate the inferior vena cava diameter during expiration and normal function of the central venous pressure (should collapse when participants take a rapid “sniff” in), and the presence of any atrial septal defects (i.e. patent foramen ovale). The suprasternal view was also obtained in the supine position with the patients head tilted backwards and the transducer placed in the sternal notch pointed inferiorly to view the long axis of the aortic arch. The aforementioned imaging windows were optimised prior to acquisition using 2D imaging optimisation techniques described in the ASE guidelines (i.e. greyscale maps, dynamic range, transmit frequency, harmonic imaging, sector size and depth). Standard imaging techniques of 2D spectral, colour and tissue Doppler were also applied as described previously [65,67]. Myocardial strain and 3D volumes and masses were also derived. Strain and strain rate measures the actual degree of myocardial deformation (cardiac mechanics and systolic ventricular function) independent of the adjacent myocardial segments and can therefore differentiate the active and passive contraction of the myocardium [67]. Strain and strain rate was derived from 2D speckle tracking echocardiography which is an angle independent method for strain and strain rate analysis. Cardiac mechanics were assessed in the three myocardial axes related to strain; longitudinal, circumferential and radial. Apical imaging was used to assess longitudinal strain, circumferential and radial strain from the short axis view. From this, global longitudinal strain was determined and a bull's eye plot was produced. Minimum frame rates for high-quality imaging acquisition (>60 frames per second) were established [65]. Three-dimensional imaging was used to assess LV and LA volumes, mass and function. The A4C was optimised with the gain and compression set at mid ranges as previously recommended [68]. The focus, time gain compensation and field of view were also optimised to maximise temporal and spatial resolution, and volume rate. Once the operator was satisfied with the image quality, then the 3D image was captured. Cine loops of all images were recorded to USB hard drive in a raw DICOM format and native data and analysed offline again by a single experienced blinded observer.

## 3 Planned statistical analysis

Statistical analyses are to be performed with SPSS 20.0 (IBM Australia Ltd, New South Wales, Australia) and STATA 11 software (StataCorp, College Station, Texas). All data will be reported as group means with confidence intervals, with default statistical significance assumed at *P* < 0.05. Cross-sectional results for each outcome measure refer to baseline entry (week 0) data. Response to training data is post minus pre for END and RES training. Normality of groups (MZ and DZ) will be assessed via Shapiro-Wilk test of normality where normality of spread is assumed at *P* > 0.05. Between-group (MZ vs DZ) differences will be assessed with the conservative non-parametric Mann-Whitney *U* test and regression models including the single covariate zygosity (MZ/DZ), fitted using a generalised estimating equation (GEE) approach to account for correlation of twins within-pairs. The effect of the exercise interventions on each outcome measure will be assessed using a linear mixed models approach.

The primary objective of the current study is to estimate the MZ and DZ correlations and the variance components A, C and E for each outcome measure cross-sectionally, and in response to exercise training (END and RES). In STATA mixed models effects will calculate intraclass correlation coefficients (r) for MZ and DZ twin pairs. Covariates to be adjusted for (e.g. sex, fat-free mass, VO_2max_, baseline data) will depend on the primary outcome measure. The subsequent rMZ and rDZ, and their respective P-values and 95% confidence intervals will be presented. Differences between intraclass correlations (rMZ vs rDZ) will be assessed using the likelihood-ratio test in STATA to compare mixed effects models constraining covariances for MZ and DZ pairs to be equal with models allowing different covariances for MZ and DZ pairs [69]. If the model allowing different MZ and DZ covariances are significantly better, and rMZ are significantly different to rDZ, A, C and E will then be calculated from the estimates of the DZ covariance and the additional MZ covariance, and confidence intervals for A, C and E will be estimated using the delta method. When rMZ and rDZ are not significantly different then a single model that includes all twin pairs but constrains the covariance to be equal for MZ and DZ pairs will be used in STATA to calculate C and E components only, and their respective 95% confidence intervals. An additional model which estimates covariance for MZ twins but fixes the DZ covariance to be zero will test if the estimated correlations suggest that the equal environments assumption has been violated.

Secondary objectives of this study will be addressed using the individual change with training (END and RES) results for each outcome measure. Response rates for each modality can be derived as a percentage of positive responses (>0) and compared against one another by a Z-test. Concordances can be derived as the percentage of individuals who positively respond to both exercise training modalities (Supplementary Fig. 5). In a similar way to the correlation analyses, within-twin pair concordances for MZ and DZ can be derived for each exercise intervention and compared to each other.

### 3.1 Washout, carryover and order effects

Analysis of the effectiveness of the washout period, carryover effects and the order effect of the exercise interventions will be assessed in SPSS and STATA. To assess the effectiveness of the washout period, the presence of carryover effects, or order effect of the exercise interventions, we will initially calculate and present summary statistics comparing groups according to the order of first administration for each intervention. The entire time series of data, including time-points following the washout, will also be presented. As a first descriptive step, group means for these comparisons will be compared using *t*-test, assuming data is normally distributed. If not, non-parametric alternatives will be used. In this way, the magnitude of change for those completing END 1st versus 2nd, and RES 1st versus 2nd, will be presented and compared. This approach will be followed by mixed models, where an ‘order’ variable (eg endurance first, endurance second) will be included in models as a factor in interactions. The length of our washout period was based on our previous findings and we do not anticipate persistent effects for the variables we will measure in this study, but any significant effects will be addressed by inclusion in our statistical models (mixed models ANOVA) of baseline values as covariates. The dependence of twins within a pair will be accounted for by fitting regression models adjusting for correlations within-twin pairs using the GEE approach in STATA to test whether time period (week 0 vs week 24) or order (END/RES vs RES/END) are associated with the outcomes.

### 3.2 Sample size and power

Power analysis is based on our primary outcome measures which are highly specialized and sensitive; vascular function (FMD), cardiac and cerebrovascular adaptation. Our cardiac publications [6,8,9] have reported significant effects in fewer subjects (n = 10 and n = 13 per group) than we recruited in the present study; we observed highly significant differences utilising highly sensitive MRI. Regarding cerebral measures, previous publications and our pilot work demonstrate that cerebral blood flow is increased in older individuals (n = 9, 65–78 yrs) by 10% after intervention. Our previous twin study publications in a paediatric population [24,31] cross-sectionally revealed FMD correlations of 0.6 and 0.38 in MZ and DZ twin pairs, respectively, in a smaller sample size of 22 (11 pairs) per group [24]. In addition, this study applied an exercise intervention (8 weeks) shorter than the current study (12 weeks), and produced correlations of 0.74 and 0.37 for FMD responses in MZ and DZ groups (n = 24–6 pairs per group) [31]. A power analysis (G*Power v3.1.2), performed using the predicted correlations for rMZ = 0.80 and rDZ = 0.30, assuming alpha = 0.05, indicates that a sample size of 23 individuals in each group provides 80% power.

## 4 Summary

Exercise is well established to confer multiple physiological, cardiovascular and mental health benefits and can therefore assist in prevention and treatment of numerous chronic diseases. However, exercise responses can be idiosyncratic, and not all individuals experience equal adaptation or health benefit from distinct exercise modalities. Currently, we do not fully understand how to optimally prescribe a personalised exercise program to elicit the greatest health and fitness benefits for any given individual, or whether there is a genetic basis for determining responsiveness to exercise.

This study aims to assess the response to two distinct forms of training. We wish to assess whether individuals who do not receive substantive health and fitness benefits from one form of exercise, will respond to another distinct form of training. The current study will also engage MZ and DZ twin pairs in exercise training to assess whether there is a genetic component to exercise training responsiveness to distinct modes of training. The results of this study have implications for prescribing personalised exercise programs to elicit the largest benefits for a given individual, and may translate to optimisation of the prevention and treatment of some chronic diseases.

## Trial registration

This trial was registered with the ANZCTR in August 2016 (registration number ACTRN12616001095459).

## Declaration of competing interest

None to declare.
