# Supplementary material for: Exploring human trainability: Design and rationale of Studies of Twin Responses to Understand Exercise as a Therapy (STRUETH) study
Source: Contemp Clin Trials Commun. 2020 Jun 9;19:100584. doi: 10.1016/j.conctc.2020.100584 (PMC7300141; doi:10.1016/j.conctc.2020.100584)
Supplement: Multimedia component 1 [file mmc1.docx]

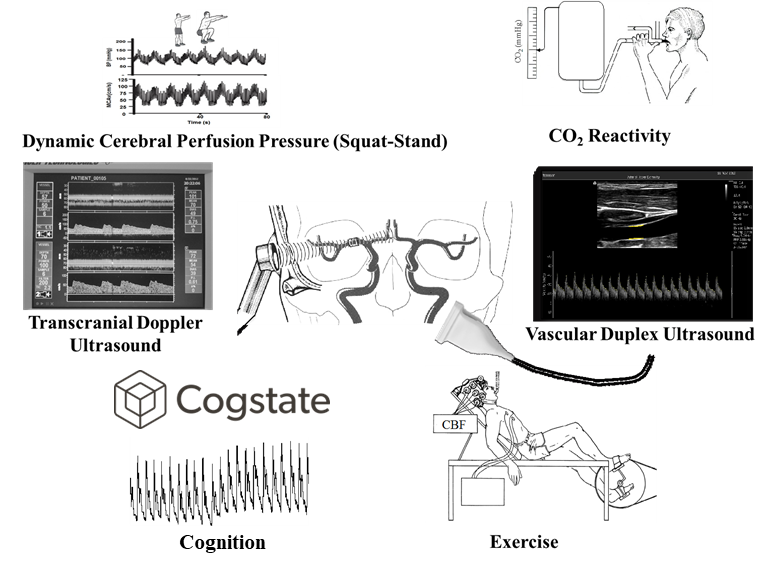


***Supplementary Figure 1.*** Schematic summary of the assessment battery of cerebrovascular measures of function and health developed by Ainslie and Green. Transcranial Doppler (TCD) measures are complemented by contemporaneous assessment of whole brain blood flow, derived from simultaneous high resolution ultrasound via insonation of the internal carotid and vertebral arteries.
