# Supplementary material for: Exploring human trainability: Design and rationale of Studies of Twin Responses to Understand Exercise as a Therapy (STRUETH) study
Source: Contemp Clin Trials Commun. 2020 Jun 9;19:100584. doi: 10.1016/j.conctc.2020.100584 (PMC7300141; doi:10.1016/j.conctc.2020.100584)
Supplement: Multimedia component 2 [file mmc2.docx]

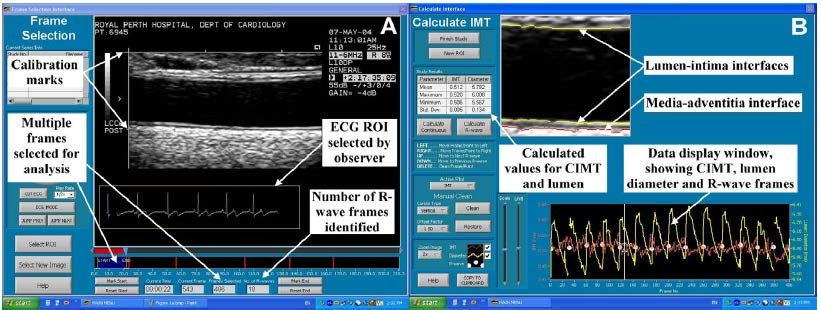


**Supplementary Figure 2.** Specialised edge-detection and wall-tracking software. Assessment of diameter, flow and shear across the cardiac cycle. Left: Diameter is calculated from ~400 measures within the region of interest at 30Hz. Velocity is also calculated via waveform envelope algorithm. Right panel: Assessment of wall thickness using validated automated algorithms.
