# Supplementary material for: Exploring human trainability: Design and rationale of Studies of Twin Responses to Understand Exercise as a Therapy (STRUETH) study
Source: Contemp Clin Trials Commun. 2020 Jun 9;19:100584. doi: 10.1016/j.conctc.2020.100584 (PMC7300141; doi:10.1016/j.conctc.2020.100584)
Supplement: Multimedia component 3 [file mmc3.docx]

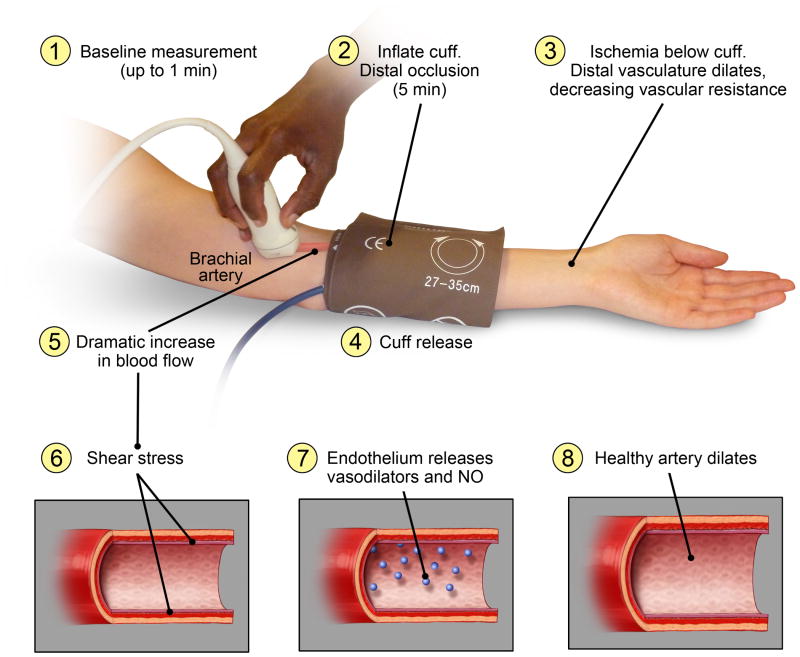


***
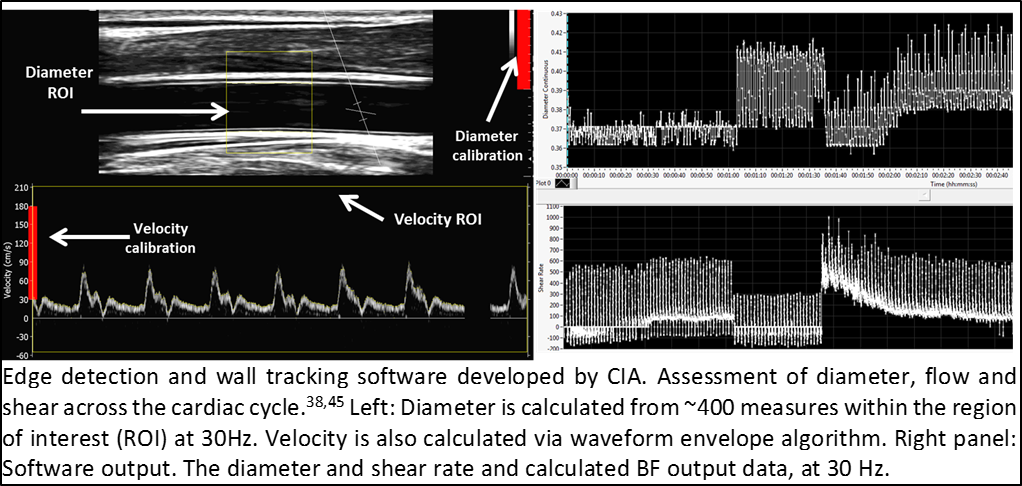
Supplementary Figure 3:*** Top: Description of flow-mediated dilation technique for in vivo assessment of conduit artery endothelium-mediated vasodilation. Edge detection and wall tracking software developed by Prof Green. Assessment of diameter, flow and shear across the cardiac cycle [1-2]. Bottom left: Diameter is calculated from >300 measures within the region of interest at 30Hz. Velocity is also calculated via waveform envelope algorithm. Bottom right: output of software – continues diameter changes and shear rate stimulus.

1. **Black MA, Cable NT, Thijssen DHJ, and Green DJ**. Impact of age, sex and exercise on brachial artery flow-mediated dilation. *Am J Physiol* 297: 1109-1116, 2009.

2. **Green DJ, Cheetham C, Mavaddat L, Watts K, Best M, Taylor RR, and O'Driscoll G**. Effect of lower limb exercise on forearm vascular function: Contribution of nitric oxide. *Am J Physiol* 283: H899-H907, 2002.
