# Supplementary material for: Exploring human trainability: Design and rationale of Studies of Twin Responses to Understand Exercise as a Therapy (STRUETH) study
Source: Contemp Clin Trials Commun. 2020 Jun 9;19:100584. doi: 10.1016/j.conctc.2020.100584 (PMC7300141; doi:10.1016/j.conctc.2020.100584)
Supplement: Multimedia component 4 [file mmc4.docx]

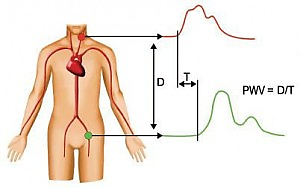


**Supplementary Figure 4.** Diagram illustrating the calculations used to quantify pulse wave velocity (PWV), where D is distance between the location of tonometry application at the carotid and femoral artery, and T is the time taken for the pressure wave from a single cardiac cycle to travel to the tonometry location.
