# Supplementary material for: Exploring human trainability: Design and rationale of Studies of Twin Responses to Understand Exercise as a Therapy (STRUETH) study
Source: Contemp Clin Trials Commun. 2020 Jun 9;19:100584. doi: 10.1016/j.conctc.2020.100584 (PMC7300141; doi:10.1016/j.conctc.2020.100584)
Supplement: Multimedia component 5 [file mmc5.docx]

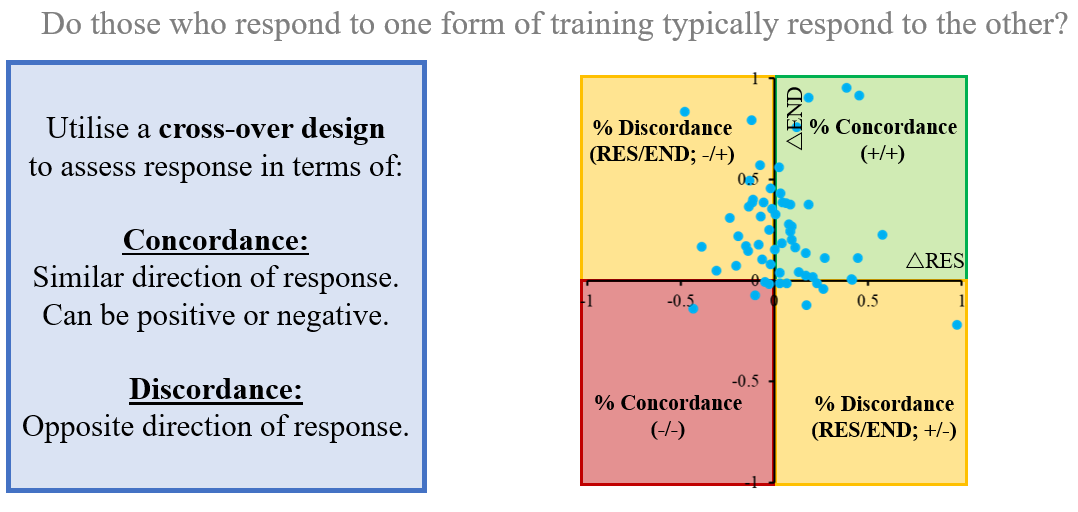


***Supplementary Figure 5.*** An example of a concordance plot (right) and descriptors of con/dis-cordance (left). In the plot, individual subject (blue dots) exercise intervention change score (△) data are plotted against one another with response to resistance (RES) and endurance (END) training on the x- and y-axis, respectively. A figure key within the plot depicts concordance and discordance for response to RES and END with percentages of responders for each quadrant reported for each variable.
